# Corrigendum to “Potential Factors for Psychological Symptoms at Three Months in Patients with Young Ischemic Stroke”

**DOI:** 10.1155/2021/9863187

**Published:** 2021-05-29

**Authors:** Dongjuan Xu, Xi Chu, Kun Wang, Lianyan Wei, Yunyun Xu, Xiaomin Huang, Jinna Li, Lina Xu, Lu Yin, Hong Liu, Xiaolei Liu, Haixia Leng, Qing Xue, Mao Peng, Longbin Jia, Hongxing Wang

**Affiliations:** ^1^Department of Neurology, Dongyang People's Hospital, Wenzhou Medical University, Zhejiang 322100, China; ^2^Health Management Department, Xuanwu Hospital, Capital Medical University, Beijing 100053, China; ^3^Department of Neurology, Beijing Puren Hospital, Beijing 100062, China; ^4^Department of Neurology, Xuanwu Hospital, Capital Medical University, Beijing 100053, China; ^5^Department of Neurology, Ningcheng Center Hospital, Inner Mongolia 024200, China; ^6^Department of Neurology, Jincheng People's Hospital, Shanxi 048026, China; ^7^Medical Research & Biometrics Centre, National Centre for Cardiovascular Diseases, Fuwai Hospital, Peking Union Medical College & Chinese Academy of Medical Sciences, Beijing, China Beijing, 102300, China; ^8^Department of Neurology, Heping Hospital Affiliated to Changzhi Medical College, Shanxi 046000, China; ^9^Department of Neurology, The First Affiliated Hospital of Kunming Medical University, Yunnan 650032, China; ^10^Beijing Psychosomatic Disease Consultation Center, Xuanwu Hospital, Capital Medical University, Beijing 100053, China; ^11^Institute of Sleep and Consciousness Disorders, Beijing Institute for Brain Disorders, Capital Medical University, Beijing 100053, China

In the article titled “Potential Factors for Psychological Symptoms at Three Months in Patients with Young Ischemic Stroke” [1], the authors identified two errors in Table 3 where “cm^3^” should be “mm^3^”. The corrected Table 3 is shown below.

## Figures and Tables

**Table 3:** 

Variables	OR (95% CI)	*P*
Model for SCL-90-R		
Family function (moderate and serious dysfunction vs. good)	2.50 (1.71, 3.54)	<0.01
Hypertension (yes vs. no)	3.27 (1.92, 4.27)	0.02
Infarct size (≥20 vs. <20 mm^3^)	2.39 (1.53, 3.45)	<0.01
Model for depression		
Marital status (single vs. married)	1.23 (1.03, 1.54)	0.01
Family function (moderate and serious dysfunction vs. good)	1.21 (1.05, 1.45)	0.03
Infarct size (≥20 vs. <20 mm^3^)	1.74 (1.14, 3.13)	0.02
Model for somatization		
Family function (moderate and serious dysfunction vs. good)	2.32 (1.51, 2.80)	<0.01
Model for anxiety		
Hypertension (yes vs. no)	2.41 (1.54, 3.46)	0.03

Abbreviations: OR: odds ratio; CI: confidence interval; SCL-90-R: Symptom Checklist 90 Revised.
